# Analytical Modeling of Riveting Squeezing Force Considering Non-Uniform Deformation of Rivets in Aeronautical Structures

**DOI:** 10.3390/ma17112756

**Published:** 2024-06-05

**Authors:** Yonggang Kang, Siren Song, Tianyu Wang, Shuaijia Kou, Guomao Li, Yonggang Chen

**Affiliations:** 1 School of Mechanical Engineering, Northwestern Polytechnical University, Xi’an 710072, China; songsiren@mail.nwpu.edu.cn (S.S.); wangtianyu1653@mail.nwpu.edu.cn (T.W.); koushuaijia@mail.nwpu.edu.cn (S.K.); guomaoli7@163.com (G.L.); scsrr11@163.com (Y.C.); 2 Xi’an Aircraft Industry Group Co., Ltd., Xi’an 710089, China

**Keywords:** riveting squeezing force, non-uniform deformation, rivet/sheet interference, barreling, Coulomb’s friction

## Abstract

Analytical modeling of the squeezing force for aircraft wings and fuselage panels in the existing literature usually assumes uniform deformation of the rivets, while in reality, the deformation of the rivets is non-uniform. To achieve high-quality squeezing force modeling, this paper introduces Coulomb’s friction and four critical adjustments to the original equation: the non-uniform rivet/sheet interference along the sheet’s hole axial ordinate; the barreling effect when calculating the driven head’s volume; the spring-back of the driven head’s dimensions; the modified height of the driven head; and the modified sheet-hole expanded diameter considering the convex structure of the driven head. The calculated values of the proposed new model demonstrate an improved level of accuracy, particularly under squeeze ratios commonly encountered in the aerospace industry.

## 1. Introduction

**Figure 1:**
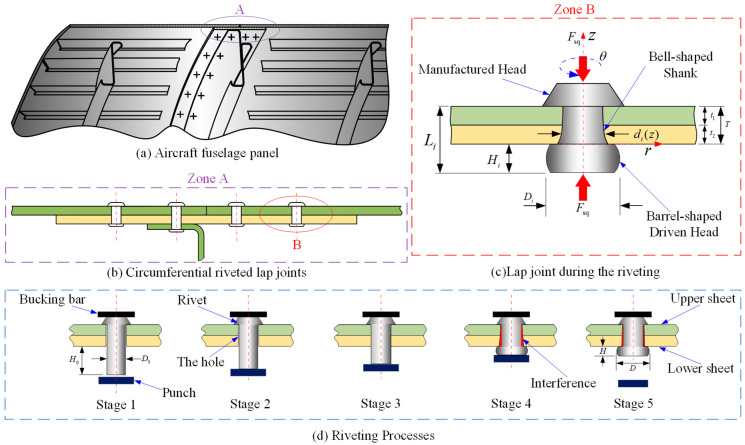
Typical riveted aircraft structure and the typical riveting process.

Aircrafts are complex systems composed of various structures, differing in materials, shapes, and dimensions [1,2,3]. Panels, created by riveting skin and stringers together, are widely used in aircraft [4,5], as illustrated in Figure 1a,b. In the riveting process, the squeezing force is one of the main setup parameters used to control the joining quality, mainly in automated riveting machines [6,7]. During riveting, a rivet undergoes non-uniform deformations, such as the driven head becoming barrel shaped driven head or the shank becoming bell shaped, as shown in Figure 1c. The squeezing force ($F_{\mathrm{sq}}$) is directly related to the driven head’s dimensions (*D_i_* and *H_i_*) and the non-uniform expanded rivet shank dimension ($d_{i}(z)$, including the interference between the rivet and the sheet hole) are depicted in Figure 1c.

Riveting involves five stages, detailed in Figure 1d, that directly influence the squeezing force, and are essential for aircrafts’ structural strength and safety [2,6]. Initially, the rivet is placed centrally in the hole between the bucking bar and punch. In stage 2, under the squeezing force, the rivet shank experiences elastic deformation, retaining a gap between the rivet shank and the sheet hole. By stage 3, plastic deformation is generated until the rivet comes into contact with the inner wall of the hole. This process involves axial compression and radial expansion of the rivet shank due to punch pressure. During stage 4, after the shank comes into contact with the inner wall of the hole, the material flow of the rivet shank inside the hole is radially constrained by the inner wall of the hole. As the squeezing force increases, the rivet shank outside the hole gradually forms a barrel-shaped driven head through friction. Simultaneously, the inside shank deforms from compression, with material from the driven head flowing into the hole. This results in non-uniform expansion of the inner wall of the hole, forming a bell-shaped rivet shank and a convex structure near the driven head. In stage 5, after maintaining the squeezing force for a certain period of time, the bucking bar and punch separate from the rivet, and the squeezing force is removed accordingly. Spring-back occurs in both the driven head and shank.

In riveting, the dimensions of the driven head determine the connection strength [3,4,5], while the interference between the rivet and the sheet hole affects the fatigue performance. Furthermore, the squeezing force primarily causes diametral expansion of the hole, leading to riveting-induced deformation or material growth [6,7,8,9,10]. Concurrently, with the aerospace industry’s widespread use of automated and robot riveting, squeezing force has emerged as a crucial parameter for riveting quality. Müller [10] was the first scholar to systematically study the riveting process, discovering the relationship between squeezing force and the dimensions of the driven head. Schijve [11], through an analytical review of Müller’s experimental data, discerned a latent correlation between the ratio of the driven head’s diameter (*D*) to its height (*H*) and the squeezing force applied. De Rijck [12,13], based on Müller and Schijve’s research, proposed a widely used model for calculating riveting squeezing force (as shown in Equations (1) and (2)) based on the assumption of the constant volume of the rivet shank.

(1) $$F_{\mathrm{sq}}=\frac{\pi }{4}D^{2}K_{r}{\left[2\ln\left(D/D_{0}\right)\right]}^{n_{r}}.$$

(2) $$F_{\mathrm{sq}}=\frac{\pi }{4}D^{2}K_{r}{\left[\ln(H_{0}/H)\right]}^{n_{r}}.$$

Figueira et al. [14,15] combined the previous studies by Müller [10], Schijve [11] and De Rijck [12,13] and introduced two factors (riveted grip thickness and friction among rivet, sheet, and punch tool) into the revised algebraic model shown in Equation (3), which is often more accurate under the medium squeeze ratio.

(3) $$\begin{array}{c} F_{\mathrm{sq}}=\frac{\pi }{2\mu^{2}}L^{2}K_{r}{\left[\ln(L_{0}/L)\right]}^{n_{r}}\left\{e^{\mu d_{2}/L}-\frac{\mu d_{2}}{L}-1\right\}\\ \;\;\;\;\;\;\;\;\;+\frac{\pi }{2\mu^{2}}H^{2}K_{r}{\left[\ln(H_{0}/H)\right]}^{n_{r}}\left\{e^{\mu (D-d_{2})/H}\left(1+\frac{\mu d_{2}}{H}\right)-\frac{\mu D}{H}-1\right\} \end{array}$$

Cheraghi [16] employed an adaptive mesh scheme, designed to counteract the non-linear characteristics of rivet materials, to simulate the riveting process. This approach significantly reduces the amount of element distortion that occurs during the driven head’s formation, thereby enhancing the accuracy of deformation estimations in the critical interface region between the sheets and the driven head. Chang et al. [17] accomplished a detailed study of riveting-induced deformation on wing panels generated by the rivet squeezing process and proposed a numerical simulation methodology based on FEM. The results obtained from the tests on a single curvature wing panel showed that there was good agreement between the experimental and simulated results. Chang et al. [18] developed a model for calculating the riveting squeezing force, which was constructed based on an analysis of the limit stress created when a thick-walled cylinder enters the plastic state. They also provided a method to solve model parameters based on the principle of a constant volume of the rivet shank. Furthermore, through numerical simulation, they validated the rationality of the division of stages in the riveting process. Zhang et al. [19] focuses on mathematically modeling and simulating the riveting process to enhance the precision of deformation analysis in thin-walled sheet metal parts, employing an ABAQUS-based calculation to analyze the stress and deformation of the model. Zeng et al. [20] derived a model of the riveting process to calculate squeezing force, and improved the model’s accuracy by taking into account the flow of rivet material into plate holes. Their model incorporates a reduction in the volume fraction of the driven rivet head and is achieved through a reduction factor determined by numerical simulation. The models of the squeezing force in the existing literature usually assume uniform deformation of the rivet, while in reality, the deformation of the rivet is non-uniform, including the deformation of the driven head and the rivet shank within the hole. This leads to an overestimation or underestimation of the squeezing force because the calculated volume and the diameter of driven head are larger, and the diameter of the rivet shank is smaller.

This study aims to propose a revised model for calculating squeezing force using the principal stress method, addressing the rivet’s non-uniform deformation. It redefines four parameter sets: the maximum expanded diameter of the sheet hole (*d*_2a_), the driven head’s equivalent diameter accounting for barrel-shape (*D*_eq_), the dimensions of the driven head after the spring-back (*D*_3_ and *H*_3_), and the modified dimensions of the expanded diameters of the driven head and sheet hole considering the convex structure of the driven head (*H*_4_ and *d*_2b_). And the purpose of the research is to identify the most critical parameter and modify the calculation equation to minimize the deviation from the experimental results.

## 2. Squeeze Force Modeling Considering the Non-Uniform Deformation of the Rivet

**Figure 2:**
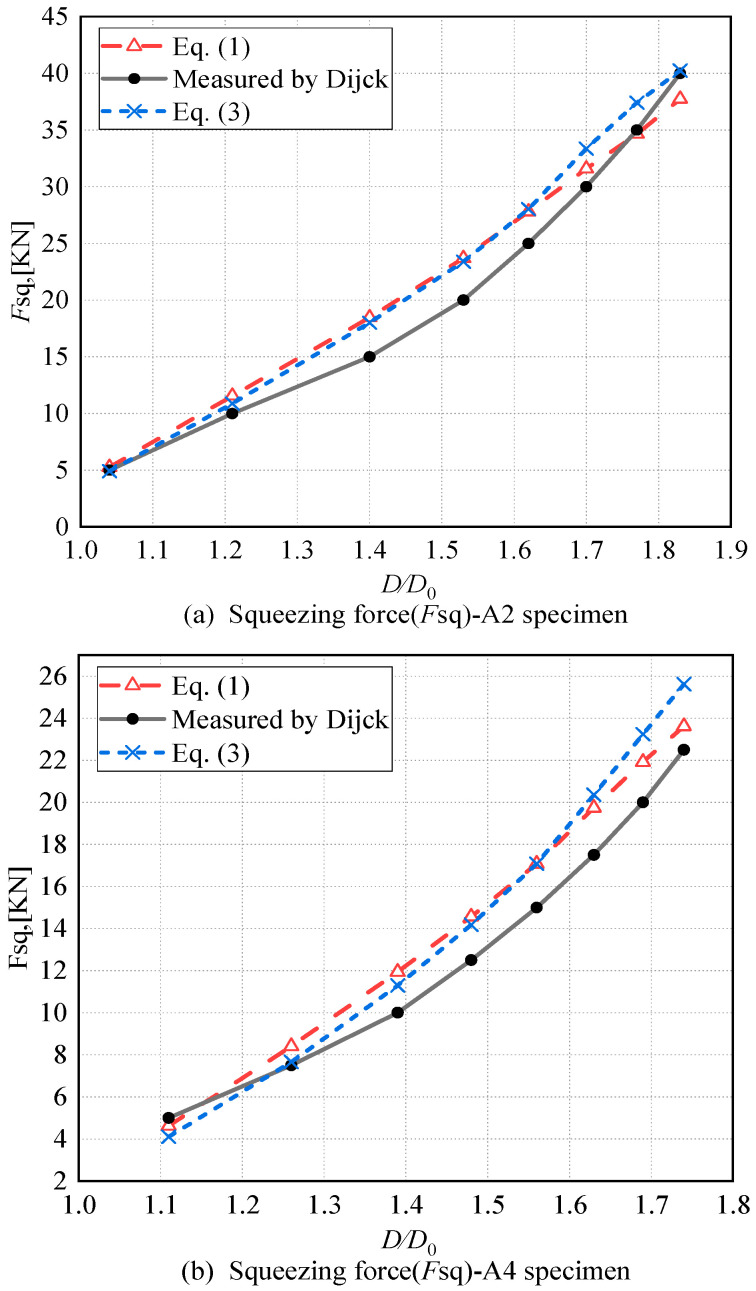
The comparison of the results obtained by Figueira et al. [15] and the results obtained by De Rijck [12] (specimens A2 and A4).

Figueira et al. [15] demonstrated that Equations (1) and (2) have an overall good agreement with experimental or FEM results. There is only a slight tendency to obtain underestimated values for high squeeze ratios (*D*/*D*_0_), which are generally above a ratio of 1.6; and in median ratios, from 1.3 up to 1.6, there is a slight tendency to obtain overestimated values, although that may not be exactly true in all cases (see Figure 2). To address this issue, Figueira et al. introduced Equation (3), which applies a strain correction for the rivet material at the hole boundaries and takes two factors (riveted grip thickness and friction among rivet, sheet, and punch tools) into consideration. However, experimental findings suggest that this correction, while helpful, is often insufficient, as the overestimation of squeezing force still occurs in many instances. Specimens A2 and A4 have the same thickness (*t*_1_ = *t*_2_ ≈ 2 mm) and use the same rivets (NAS1097D5-6), making them comparable.

**Figure 3:**
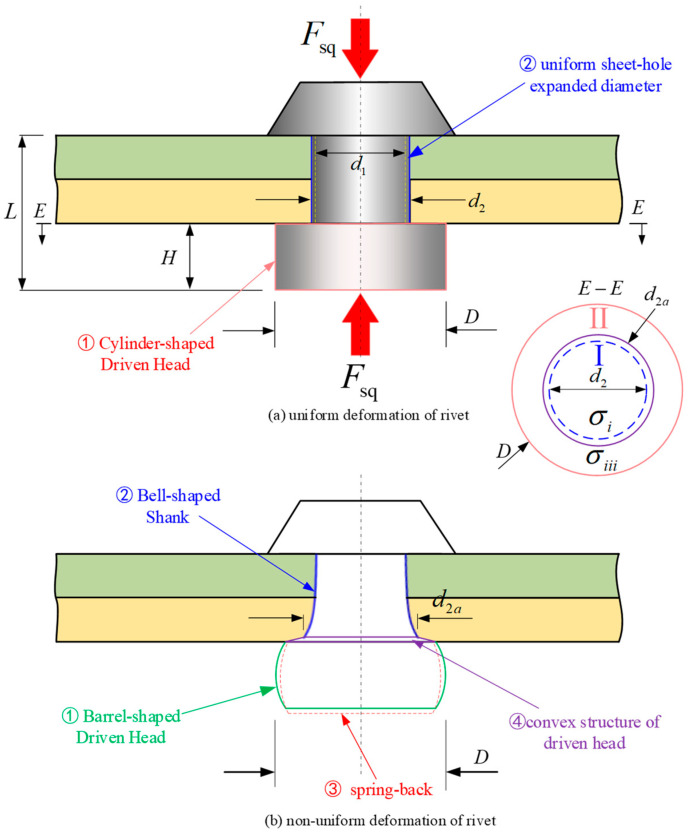
Four factors affecting the non-uniform deformation of the rivet.

The observed deviations can primarily be attributed to the fact that Equations (1)–(3) are based on the assumption of uniform deformation in rivets, as shown in Figure 3a. Contrary to this assumption, the deformation of the driven head and the shank within the rivet hole is actually non-uniform due to the effects of friction and the constraints imposed by the rivet hole, as shown in Figure 3b. Consequently, the maximum diameter of the driven head (*D*) used in Equations (1)–(3) inevitably leads to an overestimation of the squeezing force because the calculated volume of the driven head is larger than the actual barrel-shaped volume. Additionally, the integration area of stress is also larger than the actual one. Moreover, the uniform diameter (*d*_2_) of the rivet shank used in the Equation (2) is smaller than *d*_2a_ (as shown in Figure 3a). This results in an overestimation of the size of the barrel-shaped portion of the driven head (zone Ⅱ), given by (*D* − *d*_2_) > (*D* − *d*_2a_), thereby leading to an overestimation of the calculated squeezing force. This is because the stress value ($\sigma_{iii}$) in zone Ⅱ is higher than that ($\sigma_{ii}$) in zone Ⅰ due to a greater effect of strain hardening outside the shank/hole interface area [15].

Furthermore, the spring-back of the driven head, which results in an increase in diameter and a decreased height post-deformation, contributes to a reduction in the squeezing force, a factor not accounted for in Equations (1)–(3). The non-uniformity of interference leads to maximum interference on one side of the contact between the rivet hole and the driven head. In this region, the actual strain is also smaller; hence, the predicted squeezing force should be correspondingly smaller. And the spring-back of the driven head results in an increase in height and a decrease in diameter, while the material of the driven head inserted into the rivet hole leads to the formation of a convex structure near the driven head. These are factors that are not accounted for in Equations (1)–(3) which can make the calculation of squeezing force more precise. So, our study integrates the aforementioned four factors into a revised analytical model aimed at mitigating computational errors, irrespective of high, medium, or low squeeze ratios. In this section, four research tasks are delineated for execution: (1) the determination of rivet volume at various stages; (2) the squeezing force derivation using the principal stress method; (3) the calculation of the four new parameters; (4) the establishment of a new calculation model.

### 2.1. Determination of Rivet Volume at Various Stages

**Figure 4:**
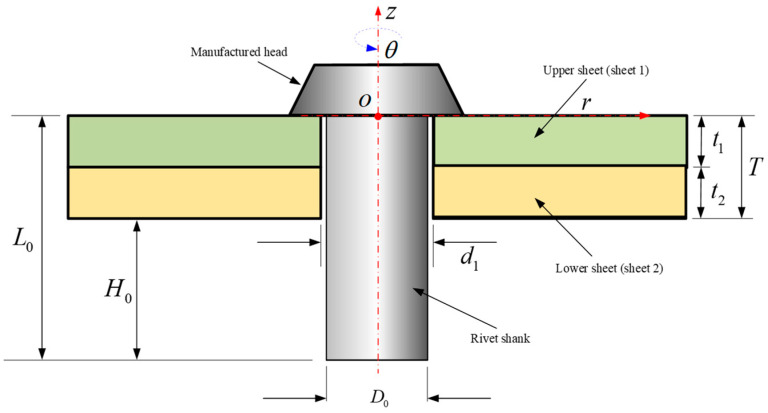
Stage 1: initial dimensions of the rivet and the polar reference coordinate system.

**Figure 5:**
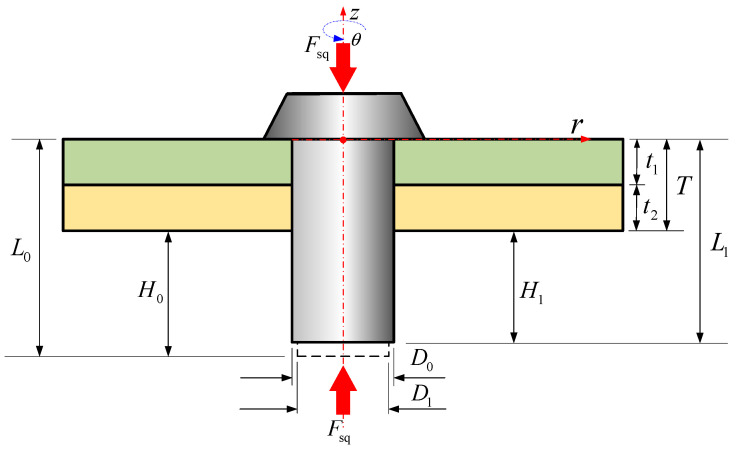
Stage 2–stage 3: the rivet shank touches the sheet hole wall under the squeezing force.

**Figure 6:**
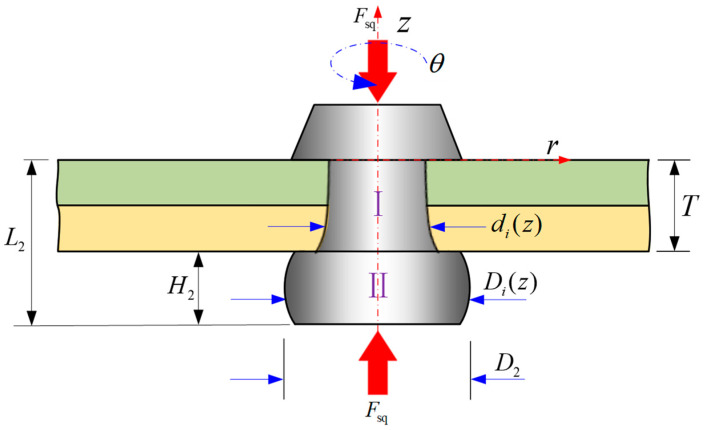
Stage 4: formation of the driven head under punching pressure.

We assume that the volume of the rivet remains constant during stages 1 to 5 of the riveting process, as depicted in Figure 3, Figure 4, Figure 5 and Figure 6. The key dimensions of the rivet, such as the height and diameter of the driven head, directly impact the calculation of squeezing force, which can be obtained by calculating the volume of the rivet shank at each stage (throughout the entire calculation process, the riveting structure is consistently assumed to be symmetrical).

The modeling process requires the adoption of a polar coordinate system (*z*, *r*, *θ*), positioned on the bottom of manufactured head, as shown in Figure 4. Within this system, the *z*-axis corresponds to the rivet shank, and its origin is located on the upper surface of the sheet 1. The model initially assumes an axisymmetric geometry for the whole model (rivet and the sheets), the volume of rivet is assumed to be constant and the thicknesses of the sheets (*t*_1_ and *t*_2_) are assumed to be kept constant. The subscript of the rivet volume *V* is standardized according to the following criteria: Arabic numerals are employed to denote the different stages of the riveting process, whereas Roman numerals are used to indicate the various components of the rivet.

In riveting stage 1, the volume of rivet shank *V*_0_ is given by

(4) $$V_{0}=\frac{\pi }{4}D_{0}^{2}L_{0}.$$

Between riveting stage 2 and riveting stage 3, under the influence of the squeezing force, the diameter of the rivet shank expands until it matches the diameter of the hole [10,11], denoted as *D*_1_. Given that the volumes in both the second and third stages are equivalent, denoted as *V*_1_, a solution is derived by concurrently solving Equations (4) and (5), leading to Equation (6). Equation (6) enables the calculation of the length of the rivet shank in stage 3, designated as *L*_1_, Ⅰ represents the volume within the hole and Ⅱ represents the volume of the driven head under punching pressure, as shown in Figure 5.

(5) $$V_{0}=\frac{\pi }{4}D_{0}^{2}L_{0}=V_{1}=\frac{\pi }{4}D_{1}^{2}L_{1},$$

(6) $$L_{1}=\frac{D_{0}^{2}L_{0}}{D_{1}^{2}}.$$

In stage 4, as illustrated in Figure 6, upon contact between the rivet and the hole wall, frictional resistance occurs at the part where the bottom of the rivet shank comes into contact with the punch, the portion of the rivet shank outside the hole gradually assumes a “barrel-like shape”, while the rivet shank inside the hole develops a non-uniform deformation, and assumes a “bell-like shape”. The volume of the rivet is denoted as *V*_2_ in stage 4, and considering that *d_i_*(*z*) is the non-uniform diameter of the rivet shank in the hole along the *z* axis, the volume of the rivet shank is *V*_Ⅰ_, and the volume of driven head is *V*_Ⅱ_ (which will be calculated in Section 2.3.1), then it can be found out that

(7) $$V_{2}=V_{Ⅰ}+V_{Ⅱ}=\frac{\pi }{4}\int_{-T}^{0}d_{i}^{2}(z)dz+\frac{\pi }{4}\int_{-L_{2}}^{-T}D_{i}^{2}(z)dz.$$

And the diameter of the rivet shank *d_i_*(*z*) is no longer constant; it is slightly larger on the driven head side at any coordinate *z.*

(8) $$d_{i}(z)\geq d_{1}.$$

**Figure 7:**
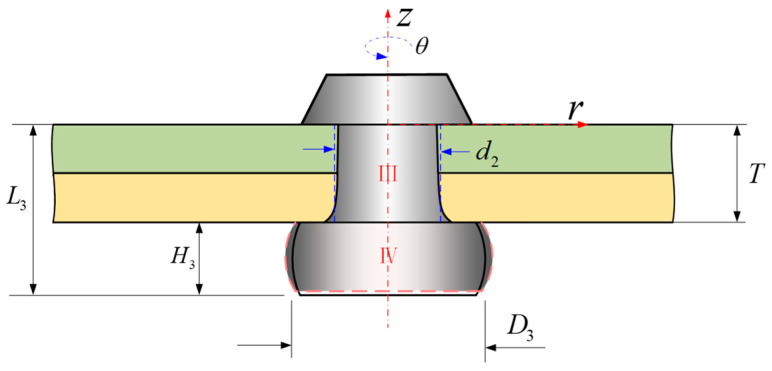
Stage 5: the squeezing force is removed; the driven head undergoes spring-back.

In Stage 5, when the final dimensions of the driven head (*D* and *H*) are reached, the squeezing force is withdrawn. Following a brief period of spring-back in both the rivet shank and the sheet hole diameters, alterations in the dimensions of the rivet ensue, Ⅲ represents the volume within the hole and Ⅳ represents the volume of the driven head undergoes spring-back, as shown in Figure 7. The volume of the rivet is denoted as *V*_3_ in stage 5, the non-uniform dimensions of rivet shank diameter is still denoted as *d_i_*(*z*), and the non-uniform driven head diameter considering the barrel-shape, *D_i_*(*z*); *V*_3_ can be expressed using Equation (9).

(9) $$V_{3}=V_{Ⅲ}+V_{Ⅳ}=\frac{\pi }{4}\int_{-T}^{0}d_{i}^{2}(z)dz+\frac{\pi }{4}\int_{-L_{3}}^{-T}D_{i}^{2}(z)dz,$$

(10) $$V_{3}=V_{2}=V_{1}=V_{0}=\frac{\pi }{4}D_{0}^{2}L_{0},$$

(11) $$\Rightarrow D_{0}^{2}L_{0}=\int_{-T}^{0}d_{i}^{2}(z)dz+\int_{-L_{3}}^{-T}D_{i}^{2}(z)dz.$$

In Figueira’s study [15], it is assumed that the interference fit during the riveting process is uniform. According to this assumption, the diameter of the rivet shank within the hole remains constant, and therefore, as shown in Figure 3a, *d*_2_ is commonly considered to be equivalent to the diameter of the rivet shank when it is pressed into the hole, based on the principle of volume constancy. And, as shown in Figure 3, we have *D*_3_ ≈ *D*_2_ = *D*, *H*_3_ ≈ *H*_2_ = *H*. Therefore, a further derivation can be obtained:

(12) $$D_{0}^{2}L_{0}=\int_{-T}^{0}d_{i}^{2}(z)dz+\int_{-L_{3}}^{-T}D_{i}^{2}(z)dz\approx d_{2}^{2}T+D^{2}H,$$

(13) $$d_{2}≅\sqrt{\frac{D_{0}^{2}L_{0}-D^{2}H}{T}}.$$

Meanwhile, it is clear that:

(14) $$H_{3}>H_{2}.$$

(15) $$D_{3}<D_{2}.$$

### 2.2. Riveting Squeezing Force Modeling

In stage 4, just before the squeezing force removal, the squeezing force *F*_sq_ can be calculated by:

(16) $$F_{\mathrm{sq}}=2\pi \int_{0}^{D_{2}/2}\sigma_{\mathrm{sq}}rdr.$$

**Figure 8:**
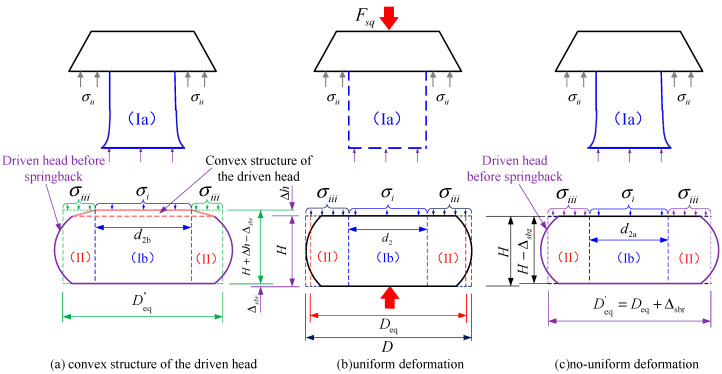
Axial stresses at rivet heads and the interface stress *σ_i_* between Zones (I_a_) and (I_b_) and *σ_iii_* (Ⅱ) (only the axial stresses).

As shown in Figure 8, it is clear that the deformation and hardening levels are different in Zone Ⅰ and Zone Ⅱ [21]. Based on the assumption of the uniform deformation of the rivet as shown Figure 8a, and introducing Coulomb’s friction condition [22,23] and the Hollomon relationship [24], the power law expression for the flow curve (e.g., Equation (17)) can be used as an approximation to predict the plastic stress–strain behavior under riveting conditions. Figueira [15] provided an adapted equation for each respective Zone’s strain condition (*σ_i_* and *σ_iii_* in Zones I_b_ and II, respectively, as shown in Figure 8a):

(17) $$\sigma_{\mathrm{sq}}(r)=\sigma_{z}=\sigma_{\mathrm{yr}}e^{\left[(2\mu /H)(D/2-r)\right]}=K_{r}{\left(\varepsilon_{\mathrm{sq}}\right)}^{n_{r}}e^{\left[(\mu /H)(D-2r)\right]}.$$

Based on the assumption of the non-uniform deformation of the rivet, as shown Figure 8b, Equation (17) needs to be rewritten as:

(18) $$\sigma_{i}=K_{r}{\left(\varepsilon_{\mathrm{sq}(Ⅰb)}\right)}^{n_{r}}e^{\left[(\mu /L_{2}^{\prime })(d_{2a}-2r)\right]}=K_{r}{\left[|\ln(L_{2}^{\prime }/L_{0})|\right]}^{n_{r}}e^{\left[(\mu /L_{2}^{\prime }(d_{2a}-2r)\right]},$$

(19) $$\sigma_{iii}=K_{r}{\left(\varepsilon_{\mathrm{sq}(Ⅱ)}\right)}^{n_{r}}e^{\left[(\mu /H_{2}^{\prime })(D_{\mathrm{eq}}^{\prime }-2r)\right]}=K_{r}{\left[|\ln(H_{2}^{\prime }/H_{0})|\right]}^{n_{r}}e^{\left[(\mu /H_{2}^{\prime })(D_{\mathrm{eq}}^{\prime }-2r)\right]},$$

In which $H_{2}^{\prime }=H_{2}-\Delta_{\mathrm{sbz}}$, $D_{\mathrm{eq}}^{\prime }=D_{\mathrm{eq}}+\Delta_{\mathrm{sbr}}$, $L_{2}^{\prime }=L_{2}-\Delta_{\mathrm{sbz}}$, $\varepsilon_{\mathrm{sq}(\mathrm{lb})}$ represent the effective plastic strain of the portion of the driven head with a diameter of *d*_2b_, *d*_2a_, *d*_2_ under different interference distributions; $\varepsilon_{\mathrm{sq}(Ⅱ)}$ represents the effective plastic strain of the remaining portion of the driven head.

Refering to Figure 8b, the squeezing force can be calculated by

(20) $$F_{\mathrm{sq}}=2\pi \int_{0}^{d_{\mathrm{eq}}^{\prime }/2}\sigma_{\mathrm{sq}}rdr=2\pi \int_{0}^{d_{2a}/2}\sigma_{i}rdr+2\pi \int_{d_{2a}/2}^{d_{\mathrm{eq}}^{\prime }/2}\sigma_{iii}rdr.$$

By simultaneously combining Equations (16)–(20), the overall expression Equation (21) can be derived

(21) $$\begin{array}{c} F_{\mathrm{sq}}=\frac{\pi }{2\mu^{2}}{L_{2}^{\prime }}^{2}K_{r}{\left[\ell_{n}(L_{0}/L_{2}^{\prime })\right]}^{n_{r}}\left\{e^{\mu d_{2a}/L_{2}^{\prime }}-\frac{\mu d_{2a}}{L_{2}^{\prime }}-1\right\}\\ \;\;\;+\frac{\pi }{2\mu^{2}}{H_{2}^{\prime }}^{2}K_{r}{\left[\ell_{n}(H_{0}/H_{2}^{\prime })\right]}^{n_{r}}\left\{e^{\mu (D_{\mathrm{eq}}^{\prime }-d_{2a})/H_{2}^{\prime }}\left(1+\frac{\mu d_{2a}}{H_{2}^{\prime }}\right)-\frac{\mu D_{\mathrm{eq}}^{\prime }}{H_{2}^{\prime }}-1\right\} \end{array}.$$

in which $H_{2}^{\prime }=H-\Delta_{\mathrm{sbz}}$, $L_{2}^{\prime }=L_{2}-\Delta_{\mathrm{sbz}}$, $D_{\mathrm{eq}}^{\prime }=D_{\mathrm{eq}}+\Delta_{\mathrm{spr}}$.

Considering the convex structure of the driven head shown in Figure 8c, the derivation process is the same as Equation (19), leading us to another model:

(22) $$\begin{array}{c} F_{\mathrm{sq}}=\frac{\pi }{2\mu^{2}}{L_{2}^{\prime }}^{2}K_{r}{\left[\ell_{n}(L_{0}/L_{2}^{\prime })\right]}^{n_{r}}\left\{e^{\mu d_{2b}/L_{2}^{\prime }}-\frac{\mu d_{2b}}{L_{2}^{\prime }}-1\right\}\\ \;\;\;+\frac{\pi }{2\mu^{2}}{H_{4}^{\prime }}^{2}K_{r}{\left[\ell_{n}(H_{0}/H_{4}^{\prime })\right]}^{n_{r}}\left\{e^{\mu (D_{\mathrm{eq}}^{\prime \prime }-d_{2b})/H_{4}^{\prime }}\left(1+\frac{\mu d_{2b}}{H_{4}^{\prime }}\right)-\frac{\mu D_{\mathrm{eq}}^{\prime \prime }}{H_{4}^{\prime }}-1\right\} \end{array}.$$

in which $H_{4}=H+\Delta h$, $H_{4}^{\prime }=H+\Delta h-\Delta_{\mathrm{sbz}}$, $D_{\mathrm{eq}}^{\prime \prime }=D_{\mathrm{eq}}+\Delta_{\mathrm{spr}}$.

There are some parameters in Equations (21) and (22) that need to be determined, including: *d*_2a_ and *D*_eq_, *d*_2b_ and ΔH, the amount of the spring-back $\Delta_{\mathrm{sbr}}$ and $\Delta_{\mathrm{sbz}}$.

To determine the abovementioned unknown parameters, there are four aspects of the research that need to be addressed in Section 2.3: (1) consider the influence of non-uniform interference fit; (2) obtain the equivalent diameter of driven head; (3) calculate the dimensions of driven head after the spring-back of the rivet; (4) modify the dimensions of driven head, and the expanded sheet hole diameter considering the convex structure of the driven head. This allows us to revise the calculation model for squeezing force, thereby enhancing the overall accuracy of the squeezing force calculation.

### 2.3. The Determination of the Unknown Parameters

#### 2.3.1. Estimation of $D_{\mathrm{eq}}$ Considering the Barrel-Shaped Effect

During the formation of the rivet head, as the riveting punch presses down, the rivet material outside the hole undergoes axial compression and radial expansion. This process is accompanied by friction (*f*) between the contact surfaces, resulting in a barrel-shaped contour on the edges of the compressed rivet head [24,25,26,27,28].

**Figure 9:**
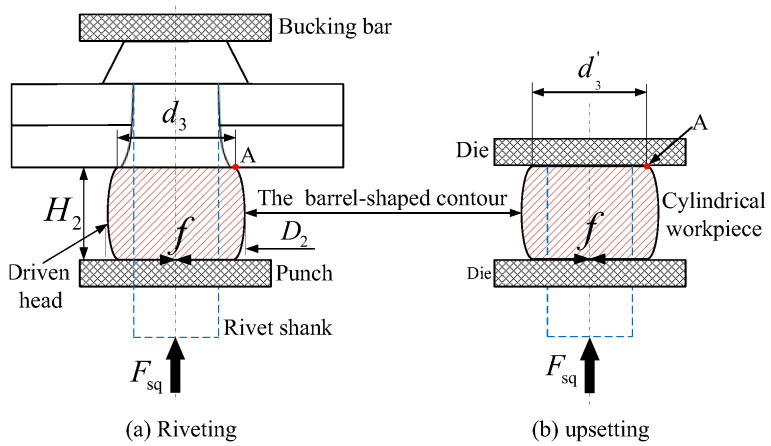
Schematic of upsetting a cylindrical forging workpiece.

As shown in Figure 9, in the barrel-shaped driven head, the key parameters include the maximum diameter *D*_2_ (which can be measured), the height of the driven head *H*_2_ (typically measurable), and the minimum diameter *d*_3_ (which needs to be calculated).

The process of the formation of a rivet driven head is similar to the upsetting process of a cylindrical workpiece, as shown in Figure 9. Therefore, under the same dimensional conditions, we equate the solution for *d*_3_ in the riveting process to the solution for $d_{3}$ in the forging process:

(23) $$d_{3}=d_{3}^{\prime }.$$

**Figure 10:**
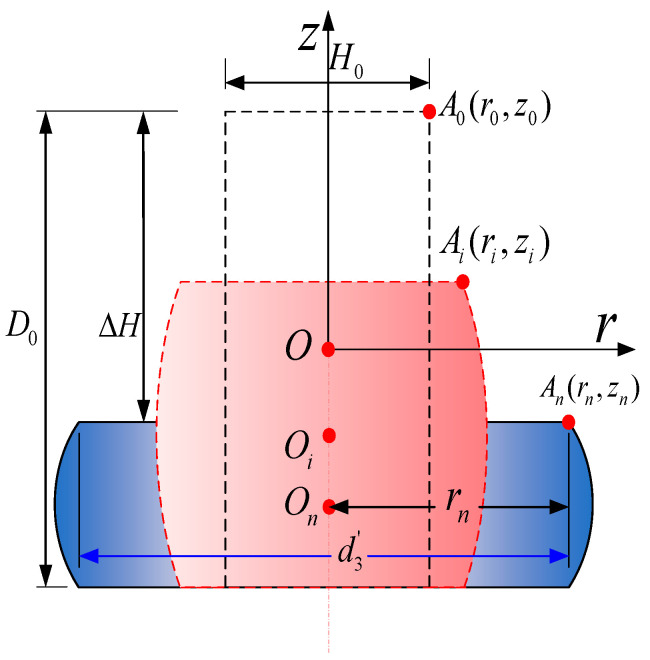
The contour change diagram of the cylindrical workpiece during upsetting process.

To obtain $d_{3}^{\prime }$, Sun [29] used the energy method to theoretically model the upsetting process of a cylindrical workpiece. They quantitatively derived the barrel-shaped contour of the deformed cylindrical metal material after upsetting through displacement increment expressions, as indicated in Equations (23)–(28). The initial calculation conditions are the coordinates (*r*_0_, *z*_0_) of any arbitrary point A on the cylindrical metal material and the final pressing amount Δ*H*. The entire pressing process is divided into *n* computational steps, as shown in Figure 10.

**Figure 11:**
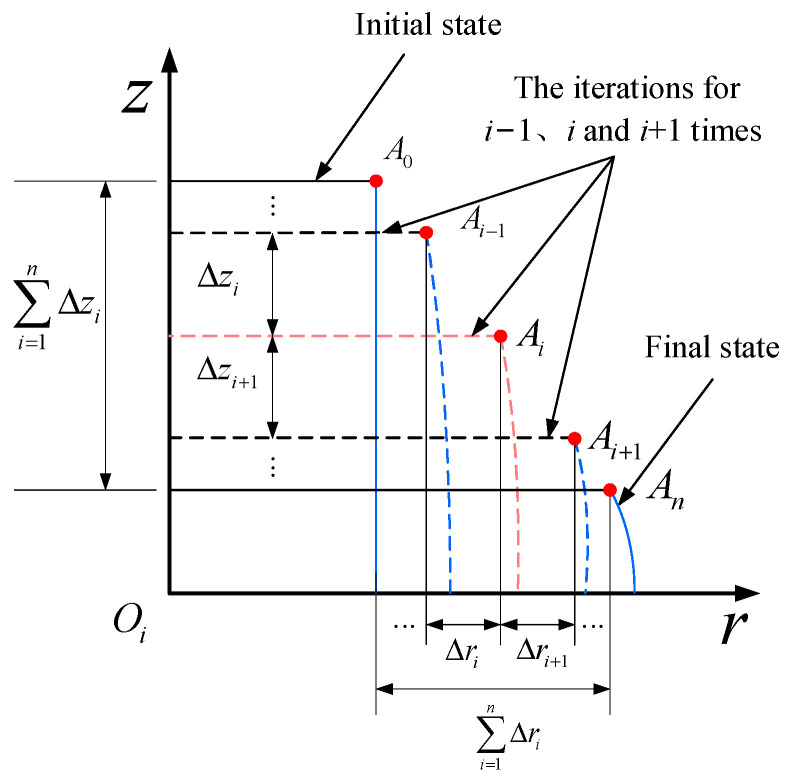
Schematic diagram of iterative solution method for contours of the cylindrical workpiece.

Using A (*r_i_*_−1_, *z_i_*_−1_) as the input, the coordinates A (*r_i_*, *z_i_*) of any point on the shape’s contour after the *i*th pressing are determined. By iterating this process, the radial displacement Δ*r* and axial displacement Δ*z* at any point on the barrel-shaped contour can be obtained, ultimately yielding the barrel-shaped contour after the upsetting deformation. In the formula, *B* is an undetermined coefficient related to friction; *H*_0_ is the initial height of the cylindrical metal material; *n* is the number of iterations; *H_i_*_−1_ is the calculated height of the cylindrical metal material after the (*i* − 1)th pressing; Δ*r_i_* is the radial displacement increment for the *i*th pressing; and Δ*z_i_* is the axial displacement increment for the *i*th pressing. The specific calculation and schematic process are illustrated in Figure 11.

(24) $$d_{3}^{\prime }=2r_{n}.$$

(25) $$\Delta r_{i}=\frac{\Delta HBr}{2H_{i-1}\sin B}\cos B\frac{z}{H_{0}},$$

(26) $$r_{n}=r_{0}+\sum_{1}^{i=n}\Delta r_{i}.$$

(27) $$\Delta Z_{i}=-\frac{\Delta H\sin B\frac{z}{H_{0}}}{\sin B},$$

(28) $$z_{n}=z_{0}+\sum_{1}^{i=n}\Delta z_{i}.$$

In these equations, *B* represents an undetermined coefficient that is associated with friction.

(29) $$B=\sqrt{6-6\sqrt{1-\frac{10\sqrt{3}\eta H_{0}/2\tau_{\max}}{3D_{0}/2\sigma_{s}}}}.$$

(30) $$\eta =\frac{1}{4}\frac{2-3c}{c{\left(1-c\right)}^{2}}+\frac{1}{5}\frac{3{\left(c\right)}^{2}-1}{c^{2}{\left(1-c\right)}^{2}}+\frac{1}{6}\frac{1-2c}{c^{2}{\left(1-c\right)}^{2}}=\frac{c-0.9c^{2}-0.2}{6c^{2}{\left(1-c\right)}^{2}},c=\frac{r_{0}}{r_{1}}.$$

where $r_{0}$ defines the location on the upper surface where the maximum frictional shear stress (*τ*_max_) occurs [30,31], which refers to the absolute distance between this point and the center of the upper surface.

(31) $$r_{1}=\frac{D_{0}}{2}.$$

(32) $$r_{0}=r_{c}=H.$$

By solving Equations (25)–(32) simultaneously, we can determine the values of *r_n_* and *z_n_*. Then, we can use Equations (24) and (26) obtain the diameter *d*_3_ of the driven head.

**Figure 12:**
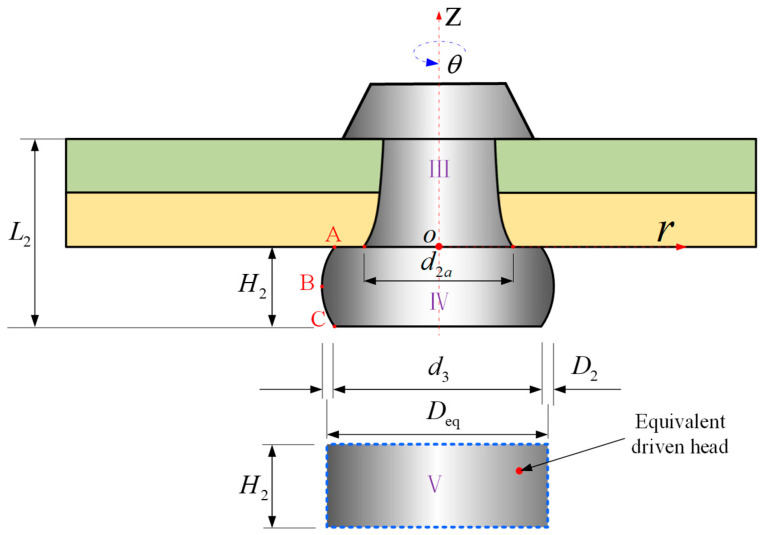
Equating the barrel-shaped volume of the driven head to the volume of a cylinder (*V*_IV_ = *V*_V_).

As shown in Figure 12, to obtain an accurate solution to $V_{\mathrm{II}}$, we assume that $d_{3}=d_{5}$. Liang et al. [32] simplified the driven head riveting process into a free upsetting procedure. Based on this simplification, a mathematical model describing the stress field of the driven head during the deformation process was proposed, followed by derivation of the driven head contour equation. Through expansion of this equation using the Taylor series, it was concluded that the external contour of the driven head model could be effectively fitted with a univariate quadratic equation.

(33) $$f\left(r\right)=Ar^{2}+Br+C.$$

In the *ZOr* coordinate system, by defining three key points, *A*, *B*, and *C,* on the outer contour of the driven head as shown in Figure 12, and by substituting the coordinate values of these points into Equation (33), the following results were obtained:

(34) $$\left\{\begin{array}{c} f\left(0\right)=C=-\frac{d_{3}}{2}\\ f\left(-\frac{H_{2}}{2}\right)=\frac{AH_{2}^{2}}{4}-\frac{BH_{2}}{2}+C=-\frac{D_{2}}{2}\\ f\left(-H_{2}\right)=AH_{2}^{2}-BH_{2}+C=-\frac{d_{3}}{2} \end{array}\right.,$$

(35) $$\left\{\begin{array}{c} A=\frac{2(D_{2}-d_{3})}{H_{2}^{2}}\\ B=\frac{2(D_{2}-d_{3})}{H_{2}}\\ C=-\frac{d_{3}}{2} \end{array}\right..$$

By performing an integral calculation on the outer contour of the driven head, we derived the volume of driven head *V*_Ⅳ_:

(36) $$V_{Ⅳ}=\pi \int_{0}^{H_{2}}{\left[f(r)\right]}^{2}dx,$$

considering Equations (31)–(33), *V*_Ⅳ_ becomes:

(37) $$V_{Ⅳ}=\frac{\pi H}{60}(8D_{2}^{2}+4d_{3}D_{2}+3d_{3}^{2}D_{2}^{2}),$$

Then, according to the principle of constant volume, the equivalent diameter of the driven head can be calculated at the same height:

(38) $$D_{\mathrm{eq}}=2\sqrt{\left(\frac{2}{15}D_{2}^{2}+\frac{1}{15}d_{3}D_{2}+\frac{1}{20}d_{3}^{2}\right)}.$$

#### 2.3.2. The Expanded Hole Diameter *d*_2a_ Considering the Non-Uniform Interference Fit

Rans and Li [33,34] used a finite element simulation to derive the typical distribution trends of rivet interference fit. They found that the radial expansion of the hole edge on sheet 1 remains relatively constant and easy to obtain:

(39) $$d_{1}\approx D_{1}.$$

**Figure 13:**
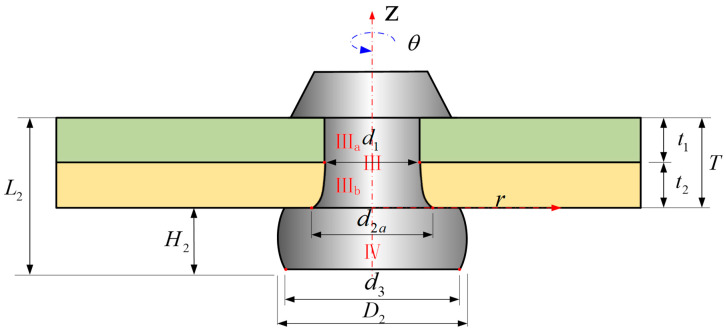
*d*_2a_ at the interface between the driven head and the lower surface of sheet 2.

So, the total volume of the rivet shank ($V_{Ⅲ}$) is assumed to consist of a cylinder ($V_{{Ⅲ}_{a}}$) and a frustum of a cone ($V_{{Ⅲ}_{b}}$), as shown in Figure 13.

(40) $$V_{{Ⅲ}_{b}}=V_{Ⅲ}-V_{{Ⅲ}_{a}},$$

(41) $$V_{0}=V_{Ⅲ}+V_{Ⅳ}=\frac{\pi }{4}D_{0}^{2}L_{0},$$

(42) $$V_{{Ⅲ}_{a}}=\pi \frac{D_{1}^{2}}{4}t_{1},$$

(43) $$V_{{Ⅲ}_{b}}=\frac{1}{12}\pi t_{2}(D_{1}^{2}+D_{1}d_{2a}+d_{2a}^{2}),$$

By combining Equations (37) and (40) through Equation (43), we can obtain *d*_2a_:

(44) $$d_{2a}=\frac{-\sqrt{\pi }D_{1}t_{2}+\sqrt{3}\sqrt{t_{2}(4\pi D_{0}^{2}L_{0}-16V_{\mathrm{IV}}-4\pi D_{1}^{2}t_{1}-\pi D_{1}^{2}t_{2})}}{2\sqrt{\pi }t_{2}}.$$

#### 2.3.3. The Influence of the Material Inserted into the Hole on the Dimensions of the Driven Head

**Figure 14:**
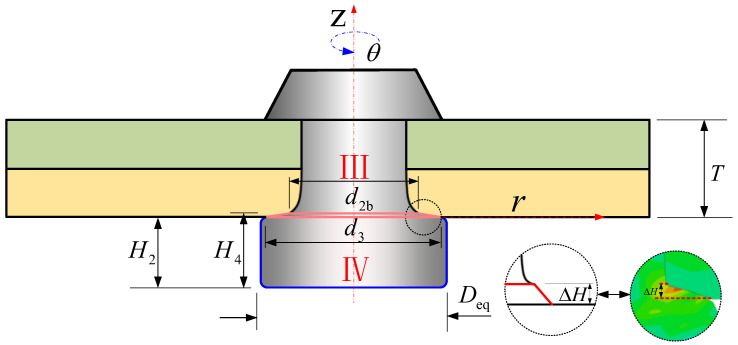
The diameter and height of the convex structure of the driven head material entering the hole.

In Equation (45), it is assumed that the volume of the rivet shank inside the hole remains unchanged [20]; however, in contrast, Equation (46) acknowledges that a portion of the driven head material is still squeezed into the hole, forming a convex structure with a height of Δ*H*, as depicted in Figure 14.

When the rivet is in contact with the hole wall, the volume of the material outside the hole is equal to the volume after the formation of the upsetting head; as such:

(45) $$V_{Ⅳ}=\frac{\pi H_{2}D_{\mathrm{eq}}^{2}}{4}+\frac{\pi \Delta H\left(d_{3}^{2}+d_{3}d_{2b}+d_{2b}^{2}\right)}{12},$$

(46) $$\frac{\pi Td_{1}^{2}}{8}=\frac{\pi (T-\Delta H)(D_{1}^{2}+D_{1}d_{2b}+d_{2b}^{2})}{24},$$

(47) $$H_{4}=H_{2}+\Delta H.$$

The solutions for Δ*H* and *d*_2b_ can be obtained through iterative calculations.

#### 2.3.4. The Spring-Back and Influence of Rivet after the Pressure Has Been Removed

During riveting stage 5, after the squeezing force has been removed, spring-back, $\Delta_{\mathrm{sbr}}$ and $\Delta_{\mathrm{sbz}}$, occurs in the driven head and shank.

**Figure 15:**
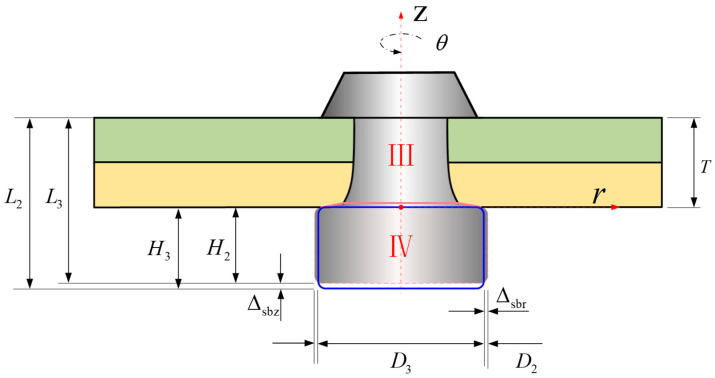
Fifth stage of riveting: the driven head final dimensions (*D*_3_ and *H*_3_) after the driven head undergoes the spring-back.

After the release of the squeezing force, the interference fit within the plate and the driven head will release their stored elastic potential energy, thereby undergoing spring-back, leading to a certain degree of volume deformation within the material, and resulting in a change in the dimensions of the driven head (*D* and *H*) (as shown in Figure 15). In stage 5, the portion of the material flowing into the rivet hole will undergo spring-back and return to the outside of the rivet hole, resulting in an increase in the volume of the driven head during this stage. In the illustration, the red area represents the diameter and height of the driven head in riveting stage 4, while the blue area represents the diameter and height of the rivet after the spring-back. The calculation of the average stress can be achieved by solving Equation (48).

(48) $$\sigma_{Ⅳ}=\frac{4F_{sq}}{\pi {D_{\mathrm{eq}}}^{2}},$$

(49) $$\varepsilon_{Ⅳz}=\frac{\sigma_{Ⅳ}}{E},$$

(50) $$\Delta_{\mathrm{spz}}=\varepsilon_{Ⅳz}L_{2}.$$

(51) $$\varepsilon_{Ⅳr}=v\varepsilon_{Ⅳr},$$

(52) $$\Delta_{\mathrm{spr}}=\varepsilon_{{Ⅳ}_{b}r}D_{\mathrm{eq}}.$$

Therefore, the driven head size considering the spring-back is

(53) $$\left\{\begin{array}{c} H_{3}=H_{4}-\Delta_{\mathrm{sbz}}\\ L_{3}=L_{4}-\Delta_{\mathrm{sbz}} \end{array}\right..$$

(54) $$D_{3}=D_{\mathrm{eq}}+\Delta_{\mathrm{sbr}}.$$

## 3. Calculation Results and Discussion

The riveting dimensions and material parameters used for analytical calculations in this paper are obtained from the following sources: the rivet parameters are derived from the relevant parameters of specimen a2 in Müller’s experimental sample [10], while the sheet material parameters are taken from MMPDS 07 [35] (as shown in Table 1). Additionally, the riveting dimensions and material parameters used for the calculations also come from De Rijck’s experimental samples A1, A2, A3, A4, and A13 [12] (as shown in Table 2).

Through experimentation, the following investigations will be carried out:

(1) Importance analysis of the proposed parameters including *d*_3_, *D*_eq_, *d*_2a_, and *S*_P_ in squeezing force calculation, aiming to identify the factor exerting the most significant influence on squeezing force calculation.
(2) Considering the non-uniform deformation of the rivet, comparisons need to be made among the calculation results obtained from Equation (21), the experimental results obtained by Müller [10] and De Rijck [12], and the calculation results obtained from Equation (3) (as shown in Table 3).
(3) Considering the convex structure of the driven head, comparisons need to be made among the calculation results obtained from Equation (22), the experimental results obtained by Müller [10] and De Rijck [12], and the calculation results obtained from Equation (3) (as shown in Table 3).

**Table 3:** Comparative of Müller’s [10] and De Rijck’s [13] experimental data and the analytical calculations based on the parameters listed in Table 1 and Table 2.

Specimens	Measured Values by Müller/De Rijck		Calculated Values			
			Equation (1)		Equation (3)	
	*D*/*D*_0_	*F*_sq_, kN	*F*_sq_, kN	Deviation, %	*F*_sq_, kN	Deviation, %
a2 (*D*_0_ = 3.208 mm; *t*_1_ = *t*_2_ = 0.83 mm; NAS1097AD4)	1.00	0.00	0.00	0.00	0.00	0.00
	1.12	4.00	3.48	−13.1	3.39	−15.3
	1.22	5.00	4.77	−4.5	4.65	−7.1
	1.27	5.53	5.57	0.8	5.45	−1.5
	1.32	6.00	6.27	4.4	6.15	2.5
	1.41	7.00	7.43	6.2	7.37	5.3
	1.45	7.55	8.14	7.8	8.12	7.5
	1.49	8.00	8.67	8.4	8.69	8.6
	1.54	9.00	9.57	6.3	9.67	7.4
	1.65	11.00	11.42	3.8	11.76	6.9
	1.66	11.10	11.64	4.9	12.02	8.3
	1.73	13.00	12.83	−1.3	13.43	3.3
	1.81	15.00	14.43	−3.8	15.41	2.8
A1 (*D*_0_ = 3.96 mm; *H*_0_ = 5.45 mm; *t*_1_ = *t*_2_ = 2.03 mm; NAS1097AD5-6)	1.00	0.00	0.00	0.00	0.00	0.00
	1.07	5.00	4.52	−9.60	4.25	−15.0
	1.18	7.50	7.43	−1.00	6.70	−10.7
	1.30	10.00	10.37	3.70	9.58	−4.2
	1.41	12.50	13.10	4.80	12.45	−0.4
	1.51	15.00	15.76	5.10	15.39	2.6
	1.57	17.50	17.61	0.60	17.56	0.3
	1.62	20.00	19.30	−3.50	19.64	−1.8
	1.68	22.50	21.07	−6.40	21.92	−2.6
A2 (*D*_0_ = 4.76 mm; *H*_0_ = 7.02 mm; *t*_1_ = *t*_2_ = 2.03 mm; NAS1097AD5-6)	1.00	0.00	0.00	0.00	0.00	0.00
	1.04	5.00	5.27	5.4	4.93	−1.40
	1.21	10.00	11.56	15.6	10.89	8.90
	1.40	15.00	18.47	23.1	18.01	20.10
	1.53	20.00	23.70	18.5	23.40	17.0
	1.62	25.00	27.80	11.2	28.03	12.1
	1.70	30.00	31.59	5.3	33.36	11.2
	1.77	35.00	34.65	−1.0	37.42	6.90
	1.83	40.00	37.73	−5.7	40.25	0.60
A3 (*D*_0_ = 5.52 mm; *H*_0_ = 9.86 mm; *t*_1_ = *t*_2_ = 3.19 mm; EN6101D7-10)	1.00	0.00	0.00	0.00	0.00	0.00
	1.10	10.00	8.40	−16.0	7.34	−26.6
	1.41	20.00	24.18	20.9	22.69	13.4
	1.52	25.00	30.40	21.6	29.28	17.1
	1.61	30.00	36.21	20.7	35.74	19.1
	1.68	35.00	40.90	16.9	41.20	17.7
	1.74	40.00	45.19	13.0	46.40	16.0
	1.79	45.00	49.54	10.1	51.93	15.4
	1.84	49.00	52.77	7.7	56.23	14.8
A4 (*D*_0_ = 3.98 mm; *H*_0_ = 5.68 mm; *t*_1_ = *t*_2_ = 2.005 mm; NAS1097D5-6)	1.00	0.00	0.00	0.00	0.00	0.00
	1.11	5.00	4.62	−7.5	4.10	−18.0
	1.26	7.50	8.41	12.1	7.66	2.1
	1.39	10.00	11.94	19.4	11.29	12.9
	1.48	12.50	14.57	16.6	14.18	13.5
	1.56	15.00	17.08	13.8	17.08	13.9
	1.63	17.50	19.74	12.8	20.36	16.4
	1.69	20.00	21.92	9.6	23.24	16.2
	1.74	22.50	23.62	5.0	25.62	13.9
A13 (*D*_0_ = 4.75 mm; *H*_0_ = 6.08 mm; *t*_1_ = *t*_2_ = 2.00 mm; MS20470AD6-6-5)	1.00	0.00	0.00	0.00	0.00	0.00
	1.02	5.00	3.47	−30.6	4.38	−12.4
	1.15	10.00	9.43	−5.7	8.81	−11.9
	1.34	15.00	16.14	7.6	15.39	2.6
	1.48	20.00	21.82	9.1	21.41	7.1
	1.59	25.00	26.07	4.3	26.20	4.8
	1.67	30.00	29.76	−0.8	30.59	2.0
	1.74	35.00	33.35	−4.7	35.11	0.3
	1.80	40.00	36.16	−9.6	38.85	−2.9

### 3.1. Analysis of Parameters’ Importance

**Figure 16:**
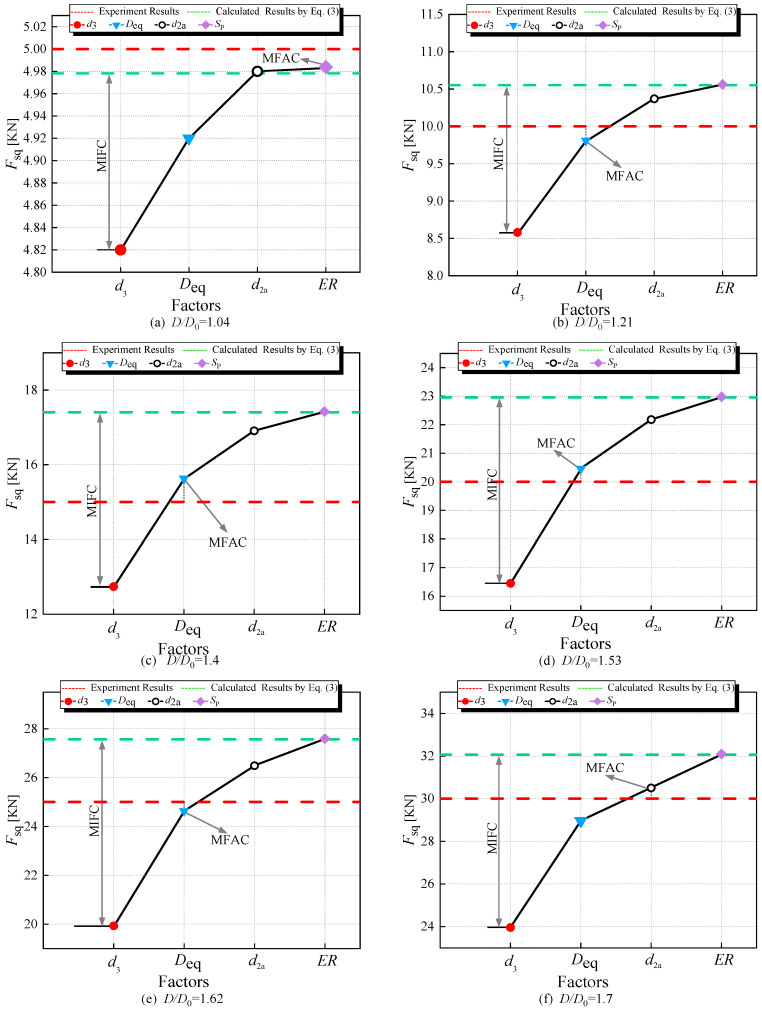

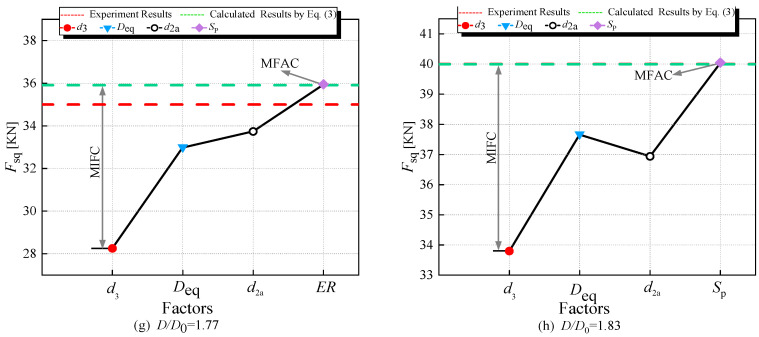
The individual effects of each factor on the squeezing force of specimen A2.

**Figure 17:**
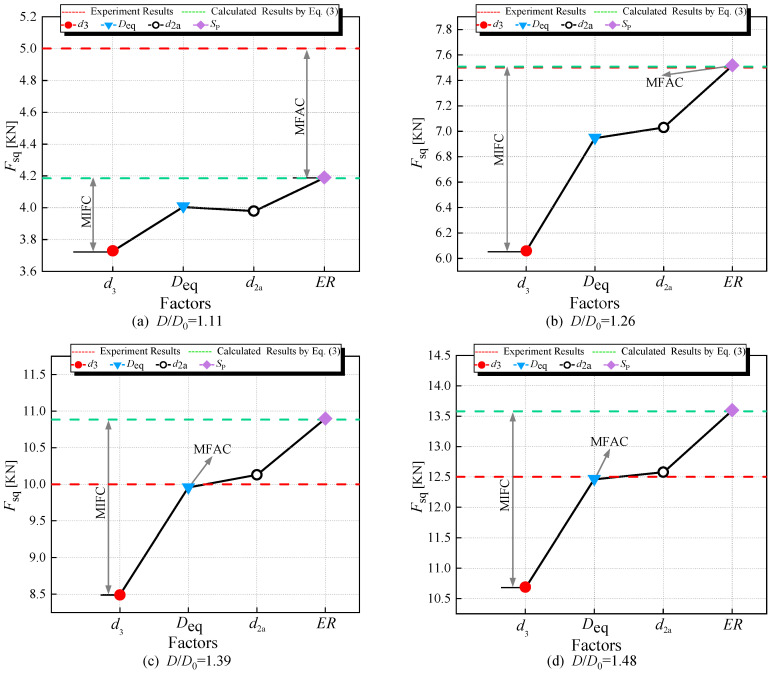

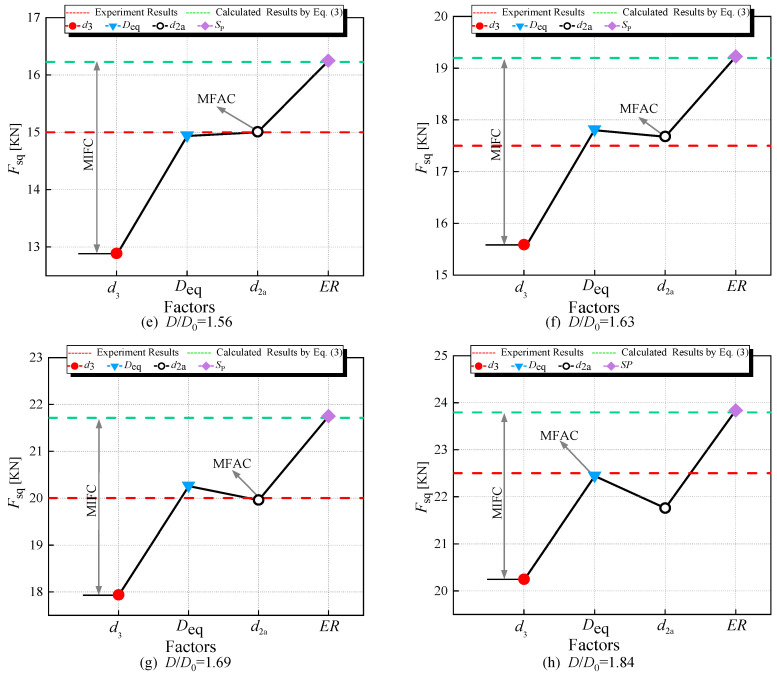
The individual effects of each factor on the squeezing force of specimen A4.

To analyze the importance of *d*_3_, *D*_eq_, *d*_2a_, and *S*_P_ in the squeezing force calculation equation, we selected specimens A2 and A4 from Table 2. Firstly, we extracted the riveting squeezing force measurement data for specimens A2 and A4 from Table 3. Then, we extracted the calculated values obtained through Equation (3), depicted, respectively, by red dashed lines and blue dashed lines in Figure 16 and Figure 17.

In Equation (3), by replacing *D* with *d*_3_ (obtained from Equation (24)), as shown in Equation (55), the calculated riveting squeezing force could be plotted as red dots; using *D*_eq_ (obtained from Equation (38)) instead of *D*, as shown in Equation (56), the resulting riveting squeezing force was plotted as blue triangles; substituting *d*_2a_ (obtained from Equation (44)) for *d*_2_, as shown in Equation (57), the resulting riveting squeezing force could be plotted as black circles; replacing *H* with *H*_3_ (obtained from Equation (53)) and *D* with *D*_3_ (obtained from Equation (54)), as shown in Equation (58), the resulting riveting squeezing force was plotted as mauve squares.

(55) $$\begin{array}{l} F_{\mathrm{sq}}=\frac{\pi }{2\mu^{2}}L^{2}K_{r}{\left[\ell_{n}(\frac{L_{0}}{L})\right]}^{n_{r}}\left\{e^{\mu d_{2}/L}-\frac{\mu d_{2}}{L}-1\right\}\\ +\frac{\pi }{2\mu^{2}}H^{2}K_{r}{\left[\ell_{n}(\frac{H_{0}}{H})\right]}^{n_{r}}\left\{e^{\mu (d_{3}-d_{2})/H}\left(1+\frac{\mu d_{2}}{H}\right)-\frac{\mu d_{3}}{H}-1\right\} \end{array}$$

(56) $$\begin{array}{l} F_{\mathrm{sq}}=\frac{\pi }{2\mu^{2}}L^{2}K_{r}{\left[\ell_{n}(\frac{L_{0}}{L})\right]}^{n_{r}}\left\{e^{\mu d_{2}/L}-\frac{\mu d_{2}}{L}-1\right\}\\ \;\;\;+\frac{\pi }{2\mu^{2}}H^{2}K_{r}{\left[\ell_{n}(\frac{H_{0}}{H})\right]}^{n_{r}}\left\{e^{\mu (D_{\mathrm{eq}}-d_{2})/H}\left(1+\frac{\mu d_{2}}{H}\right)-\frac{\mu D_{\mathrm{eq}}}{H}-1\right\} \end{array}$$

(57) $$\begin{array}{l} F_{\mathrm{sq}}=\frac{\pi }{2\mu^{2}}L^{2}K_{r}{\left[\ell_{n}(\frac{L_{0}}{L})\right]}^{n_{r}}\left\{e^{\mu d_{2a}/L}-\frac{\mu d_{2a}}{L}-1\right\}\\ \;\;\;+\frac{\pi }{2\mu^{2}}H^{2}K_{r}{\left[\ell_{n}(\frac{H_{0}}{H})\right]}^{n_{r}}\left\{e^{\mu (D-d_{2a})/H}\left(1+\frac{\mu d_{2a}}{H}\right)-\frac{\mu D}{H}-1\right\} \end{array}$$

(58) $$\begin{array}{l} F_{\mathrm{sq}}=\frac{\pi }{2\mu^{2}}L^{2}K_{r}{\left[\ell_{n}(\frac{L_{0}}{L})\right]}^{n_{r}}\left\{e^{\mu d_{2}/L}-\frac{\mu d_{2}}{L}-1\right\}\\ \;\;\;+\frac{\pi }{2\mu^{2}}{H_{3}}^{2}K_{r}{\left[\ell_{n}(\frac{H_{0}}{H})\right]}^{n_{r}}\left\{e^{\mu (D_{3}-d_{2})/H_{3}}\left(1+\frac{\mu d_{2}}{H_{3}}\right)-\frac{\mu D_{3}}{H_{3}}-1\right\} \end{array}$$

In Figure 16 and Figure 17, we must find out the major factors influencing the accuracy of the calculation (MFAC), and the major factors influencing the calculation results (MIFC).

Comparing with the experimental data and the calculated results, that the following can be seen:

(1) For A2 and A4, in the majority of cases, substituting *d*_3_ or *D*_eq_ with *D*, or *d*_2a_ with *d*_2_, or applying *S*_P_ to adjust the dimensions *D* and *H* of the driven head, resulting in a squeezing force calculation that is smaller than the computed value.
(2) In Figure 16, *d*_3_ emerges as the major influencing factor for the calculation results (MIFC), while *D*_eq_ is the major factor influencing the accuracy of the calculation (MFAC).
(3) In Figure 17, *d*_3_ remains the MIFC, whereas *d*_2_ appears to be the MFAC.
(4) Additionally, it is easy to observe from Figure 16 and Figure 17 that, relative to the experimental values, the riveting force calculated using *D*_eq_ is more accurate than that obtained using *d*_3_. Therefore, in the following calculation process, *D*_eq_ is chosen as the calculation region for *σ_iii_*.

### 3.2. Considering Non-Uniform Deformation of the Rivet

**Figure 18:**
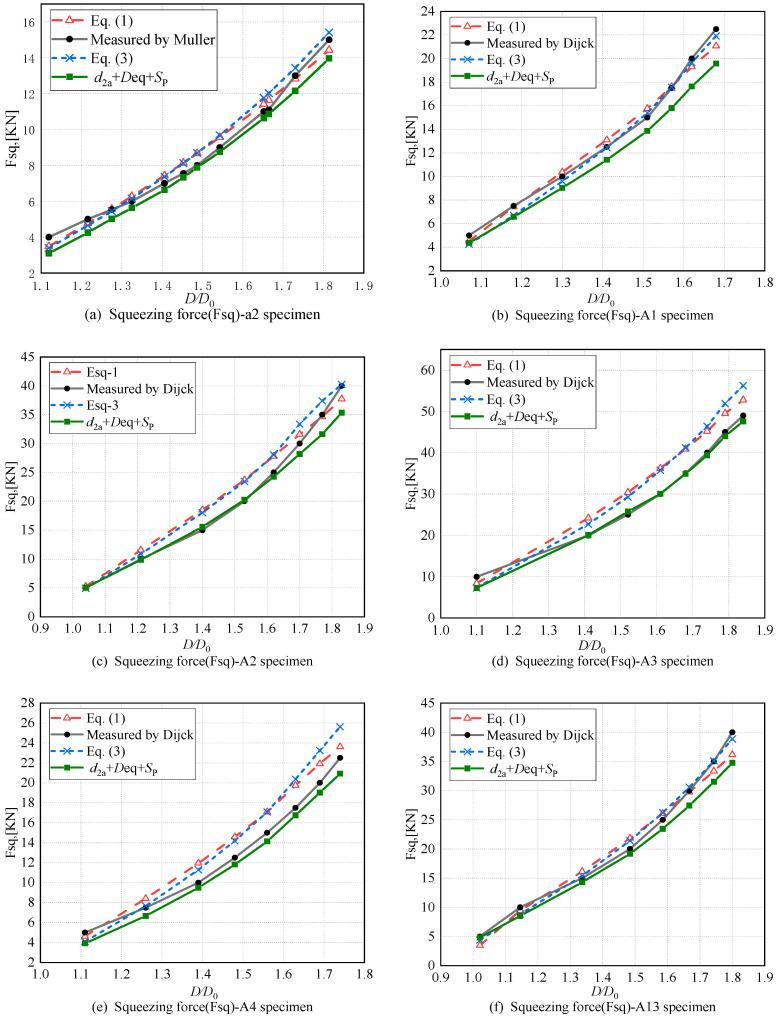
The calculated results based on the combined factors of non-uniform interference *d*_2a_ and equivalent driven head diameter *D*_eq_, and the spring-back.

Taking into account the non-uniform deformation of the rivet, the calculated squeezing force values obtained from Equation (21) are shown in Table 4. Figure 18 is plotted to compare Equations (1), (3) and (21), and the experimental data, aiming to analyze the accuracy of the predicted squeezing force calculated from Equation (21), as shown in Table 4.

**Figure 19:**
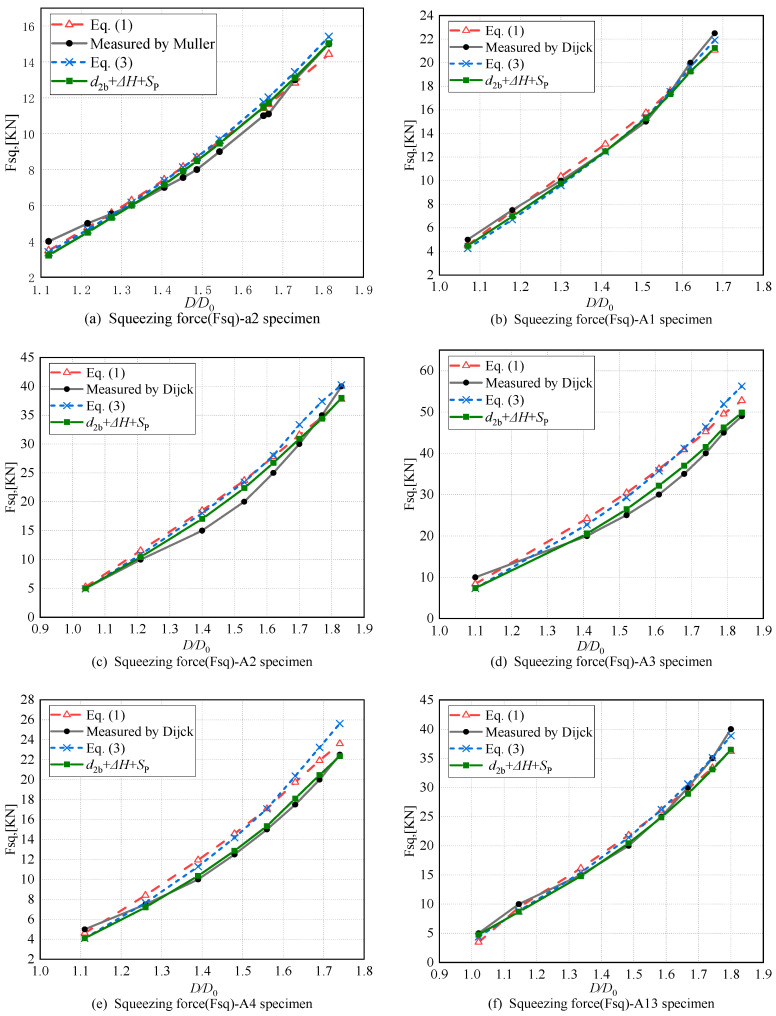
The calculated results based on the combined factors of non-uniform interference *d*_2b_, the height of convex structure Δ*H*, and the spring-back.

The overall trend in the squeezing force obtained from Equation (21) is consistent with the experimental values, but in most cases, it slightly underestimates the experimental results. The standard deviations for specimens a2, A1, A2, A3, A4, and A13 are 0.53%, 2.24%, 3.73%, 17.66%, 7.6%, and 11.16%, respectively. In Figure 18a, for specimen A2, within the medium squeeze ratio range (1.4 ≤ *D*/*D*_0_ ≤ 1.7), the predicted values from Equation (21) are more accurate compared to Equations (1) and (3), with a deviation of less than 5.28%. For specimen A4 (see Figure 18e), within the medium and high squeeze ratio range (1.39 ≤ *D*/*D*_0_), the accuracy of predictions from Equation (21) is higher compared to Equations (1) and (3), with a deviation from the experimental values of 7.03%. For specimens A1 (see Figure 18b) and A13 (see Figure 18f), overall, the predictions from Equation (21) are lower than the experimental values, but the deviation is still within 13.02%. Particularly for specimen A2 (see Figure 18c) at low to medium squeeze ratios (*D*/*D*_0_ < 1.7), and specimen A3 (see Figure 19d) at medium and high squeeze ratios (1.4 ≤ *D*/*D*_0_), the accuracy of predictions from Equation (21) is higher compared to Equations (1) and (3). The maximum deviation between predicted and experimental values is less than 4%, with deviations of 3.59% and 2.97%, respectively.

This is the case because Equations (1) and (3) use the maximum rivet head diameter *D*, which results in an overestimation due to the larger volume of the rivet head. In contrast, the predictions from Equation (21) adequately consider the influence of the barrel-shaped of the rivet head on the calculation results, resulting in an improved accuracy.

### 3.3. Considering the Effect of the Material Inserted into the Hole on the Height of the Driven Head

Considering the convex structure of the driven head, the squeezing force values calculated using Equation (22) are shown in Table 4. Subsequently, Figure 19 was be plotted to compare Equations (1), (3) and (22) with the experimental data, aiming to analyze the accuracy of the squeezing force predicted by Equation (22) as shown in Table 4. The overall trend in the squeeze force obtained from Equation (22) is consistent with the experimental values. The standard deviations for specimens a2, A1, A2, A3, A4, and A13 are 8.46%, 3.32%, 1.32%, 6.87%, 4.27%, and 1.34%, respectively. For specimen A1 (see Figure 19b), under medium squeeze ratios (1.3 ≤ *D*/*D*_0_ ≤ 1.62), Equation (22) shows a significant improvement in the overall accuracy, with deviations less than 3.77%. For specimen A13 (see Figure 19f), the prediction accuracy of Equation (22) has also improved, with deviations below 5.45% under medium-to-high squeeze ratios (1.4 ≤ *D*/*D*_0_ ≤ 1.74). For specimen a2 (see Figure 19a), under medium-to-high squeeze ratios (1.27 ≤ *D*/*D*_0_), the deviation between the calculated values from Equation (22) and the experimental values is within 5.98%. For specimen A2 (see Figure 19e), at low squeeze ratios (*D*/*D*_0_ ≤ 1.3), the accuracy of Equation (22) is higher compared to that of Equations (1) and (3), with deviations from the experimental values of 13.49%.

This improvement is attributed to considering the material inserted into the rivet hole when correcting the driven head size, resulting in a closer match to experimental results.

**Table 4:** Results calculated by four improving factors.

Specimens	Calculated Values							
	Values Measured by Müller/De Rijck		Equation (1)		*d*_2_ *+ D*_eq_ *+ ER* (Equation (21))		*ΔH + d*_2b_ + *ER* (Equation (22))	
	*D∕D*_0_	*F*_sq_, kN	*F*_sq_	Deviation, %	*F*_sq_	Deviation, %	*F*_sq_	Deviation, %
a2 (*D*_0_ = 3.208 mm; *t*_1_ = *t*_2_ = 0.83 mm; NAS1097AD4)	1.00	0.00	0.00	0.00	0.00	0.00	0.00	0.00
	1.12	4.00	3.48	−13.1	3.10	−22.44	3.22	−19.41
	1.22	5.00	4.77	−4.5	4.25	−15.04	4.50	−9.99
	1.27	5.53	5.57	0.8	5.02	−9.27	5.32	−3.72
	1.32	6.00	6.27	4.4	5.63	−6.22	6.00	0.03
	1.41	7.00	7.43	6.2	6.63	−5.28	7.17	2.49
	1.45	7.55	8.14	7.8	7.33	−2.98	7.91	4.82
	1.49	8.00	8.67	8.4	7.88	−1.51	8.48	5.98
	1.54	9.00	9.57	6.3	8.75	−2.83	9.46	5.06
	1.65	11.00	11.42	3.8	10.63	−3.33	11.47	4.28
	1.66	11.10	11.64	4.9	10.86	−2.12	11.73	5.65
	1.73	13.00	12.83	−1.3	12.15	−6.57	13.12	0.96
	1.81	15.00	14.43	−3.8	13.96	−6.95	15.03	0.22
A1 (*D*_0_ = 3.96 mm; *H*_0_ = 5.45 mm; *t*_1_ = *t*_2_ = 2.03 mm; NAS1097AD5-6)	1.00	0.00	0.00	0.00	0.00	0.00	0.00	0.00
	1.07	5.00	4.52	−9.60	4.36	−12.78	4.47	−10.57
	1.18	7.50	7.43	−1.00	6.57	−12.38	6.97	−7.10
	1.30	10.00	10.37	3.70	9.02	−9.85	9.76	−2.42
	1.41	12.50	13.10	4.80	11.41	−8.73	12.48	−0.15
	1.51	15.00	15.76	5.10	13.86	−7.59	15.29	1.93
	1.57	17.50	17.61	0.60	15.80	−9.69	17.33	−0.97
	1.62	20.00	19.30	−3.50	17.63	−11.86	19.25	−3.77
	1.68	22.50	21.07	−6.40	19.57	−13.02	21.25	−5.55
A2 (*D*_0_ = 4.76 mm; *H*_0_ = 7.02 mm; *t*_1_ = *t*_2_ = 2.03 mm; NAS1097AD5-6)	1.00	0.00	0.00	0.00	0.00	0.00	0.00	0.00
	1.04	5.00	5.27	5.4	5.06	1.16	5.00	0.08
	1.21	10.00	11.56	15.6	9.85	−1.53	10.44	4.36
	1.40	15.00	18.47	23.1	15.54	3.59	17.02	13.49
	1.53	20.00	23.70	18.5	20.25	1.25	22.35	11.74
	1.62	25.00	27.80	11.2	24.24	−3.04	26.74	6.96
	1.70	30.00	31.59	5.3	28.18	−6.08	30.91	3.05
	1.77	35.00	34.65	−1.0	31.62	−9.66	34.38	−1.76
	1.83	40.00	37.73	−5.7	35.35	−11.61	37.99	−5.04
A3 (*D*_0_ = 5.52 mm; *H*_0_ = 9.86 mm; *t*_1_ = *t*_2_ = 3.19 mm; EN6101D7-10)	1.00	0.00	0.00	0.00	0.00	0.00	0.00	0.00
	1.10	10.00	8.40	−16.0	7.31	−26.88	7.38	−26.24
	1.41	20.00	24.18	20.9	20.07	0.37	20.53	2.67
	1.52	25.00	30.40	21.6	25.74	2.97	26.41	5.62
	1.61	30.00	36.21	20.7	30.06	0.19	32.14	7.12
	1.68	35.00	40.90	16.9	34.90	−0.28	36.97	5.64
	1.74	40.00	45.19	13.0	39.38	−1.56	41.50	3.75
	1.79	45.00	49.54	10.1	44.01	−2.19	46.21	2.68
	1.84	49.00	52.77	7.7	47.61	−2.84	49.83	1.70
A4 (*D*_0_ = 3.98 mm; *H*_0_ = 5.68mm; *t*_1_ = *t*_2_ = 2.005 mm; NAS1097D5-6)	1.00	0.00	0.00	0.00	0.00	0.00	0.00	0.00
	1.11	5.00	4.62	−7.5	3.91	−21.72	4.10	−17.94
	1.26	7.50	8.41	12.1	6.66	−11.16	7.21	−3.93
	1.39	10.00	11.94	19.4	9.49	−5.06	10.36	3.56
	1.48	12.50	14.57	16.6	11.81	−5.50	12.87	2.96
	1.56	15.00	17.08	13.8	14.13	−5.80	15.35	2.34
	1.63	17.50	19.74	12.8	16.75	−4.28	18.11	3.48
	1.69	20.00	21.92	9.6	19.02	−4.92	20.46	2.29
	1.74	22.50	23.62	5.0	20.92	−7.03	22.36	−0.63
A13 (*D*_0_ = 4.75 mm; *H*_0_ = 6.08 mm; *t*_1_ = *t*_2_ = 2.00 mm; MS20470AD6-6-5)	1.00	0.00	0.00	0.00	0.00	0.00	0.00	0.00
	1.02	5.00	3.47	−30.6	4.82	−3.58	4.77	−4.60
	1.15	10.00	9.43	−5.7	8.52	−14.77	8.67	−13.33
	1.34	15.00	16.14	7.6	14.29	−4.74	14.78	−1.43
	1.48	20.00	21.82	9.1	19.16	−4.19	20.44	2.19
	1.59	25.00	26.07	4.3	23.45	−6.21	24.85	−0.61
	1.67	30.00	29.76	−0.8	27.43	−8.57	28.92	−3.60
	1.74	35.00	33.35	−4.7	31.52	−9.95	33.09	−5.45
	1.80	40.00	36.16	−9.6	34.75	−13.12	36.46	−8.85

## 4. Conclusions

A new analytical model for calculating the riveting squeezing force has been proposed, taking into account the non-uniform deformation of the rivet, rather than the uniform deformation that was previously used in the literature, in which four sets of parameters are redefined, including maximum expanded sheet hole diameter (*d*_2a_); the equivalent diameter of the driven head considering the barrel shape (*D*_eq_); the dimensions of driven head after spring-back (*D*_3_ and *H*_3_); the modified height of driven head (*H*_4_); and the modified expanded sheet hole diameter (*d*_2b_) after considering the convex structure of the driven head.

The analysis leads to the following points:

(1) The introduction of both *d*_2a_ and *D*_eq_ can reduce the calculated values of the riveting force, while the introduction of spring-back can slightly increase the calculated values of the riveting force compared to the previous model, as shown in Equation (3).
(2) The new analytical models for calculating riveting squeezing force, represented by Equations (21) and (22), which take into account the non-uniform deformation of the rivet, significantly reduced the deviation between the calculated squeezing force and experimental data, especially in the medium squeeze ratio range (1.3 ≤ *D*/*D*_0_ ≤ 1.6). The previous formulation (Equations (1) and (3)) generally overestimates the required squeezing force due to an overestimation of the true strain of the rivet material within the hole vicinity. Equations (21) and (22) significantly improved the prediction accuracy, and the deviation from the experimental values is reduced to within 10% for some specimens (while the deviation for Equations (1) and (3) sometimes exceed 20%), this is because the non-uniform deformation of the rivet is considered fully.
(3) After considering the convex structure of the driven head, the calculation accuracy is significantly improved, and most importantly, the consistency of the predicted squeezing force is improved, with a standard deviation of 8.46%. However, Equation (22) had a standard deviation of 17.66%.
(4) For other types of sheet materials, such as CFRP, and different fasteners like headless rivets, the proposed model requires modifications to maintain accuracy. And, the current study did not account for the friction between the rivet shank and the hole. To further the research in this area, the proposed model must take into account the friction that occurs between the rivet shank and the holes. This improvement would help reduce the tendency to underestimate the riveting forces in the low squeeze ratio range.

## Figures and Tables

**Table 1:** Rivet material parameters for the analytical calculations based on Muller’s experimental datal [10] and sheet material parameters based on MMPDS 07 [35].

Data	Value
Coulomb’s friction coefficient *μ* (assumed value by default)	0.15
Strength coefficient *K* for sheet material Al 2024-T3	462 Mpa
Strain hardening coefficient *n* for sheet material Al 2024-T3	0.0769
AL 2024-T3 Young’s modulus *E*	73,773.9 MPa
AL 2024-T3 compression yield stress	255.1 MPa
AL 2024-T3 Poisson’s ratio	0.33
Specimen a2: 2024-T3 bare; *t*_1_ = *t*_2_ = 0.83 mm; *D*_0_ = 3.208 mm; *H*_0_ = 4.8 mm; *d*_1_ = 3.296 mm; rivet type: NAS1097AD4	

**Table 2:** Rivet material parameters used in analytical calculations based on De Rijck’s [12] and De Rijck et al. [13] experimentally measured data or assumed by default.

Data	Value
Coulomb’s friction coefficient *μ* (assumed value by default)	0.15
Strength coefficient *K_r_* for rivet material AI 2117-T4 (AD)	600 MPa
Strength coefficient *K_r_* for rivet material Al 2017-T4 (D)	600 MPa
Strain hardening coefficient *n_r_* for rivet material Al 2117-T4 (AD)	0.30
Strain hardening coefficient *n_r_* for rivet material Al 2017-T4 (D)	0.45
Specimen A1: 2024-T3 clad; *t*_1_ = *t*_2_ = 2.04 mm; *D*_0_ = 3.96 mm; *H*_0_ = 5.45 mm; *d*_1_ = 4.10 mm; rivet type: NAS1097AD5-6	
Specimen A2: 2024-T3 clad; *t*_1_ = *t*_2_ = 2.03 mm; *D*_0_ = 4.76 mm; *H*_0_ = 7.02 mm; *d*_1_ = 4.90 mm; rivet type: NAS1097AD6-7	
Specimen A3: 2024-T3 clad; *t*_1_ = *t*_2_ = 3.18 mm; *D*_0_ = 5.52 mm; *H*_0_ = 9.86 mm; *d*_1_ = 5.60 mm; rivet type: EN6101D7-10	
Specimen A4: 2024-T3 clad; *t*_1_ = *t*_2_ = 2.01 mm; *D*_0_ = 3.98 mm; *H*_0_ = 5.68 mm; *d*_1_ = 4.10 mm; rivet type: NAS1097D5-6	
Specimen A13: 2024-T3 clad; *t*_1_ = *t*_2_ = 2.00 mm; *D*_0_ = 4.75 mm; *H*_0_ = 6.08 mm; *d*_1_ = 4.90 mm; rivet type: MS20470AD6-6-5	

## Data Availability

The original contributions presented in the study are included in the article, further inquiries can be directed to the corresponding author.

## Nomenclature

D	the diameter of installed driven rivet head (or tail head diameter)
$D_{0}$	the diameter of uninstalled or original rivet shank
$D_{1}$	the diameter of rivet shank when shank touching sheet-hole wall (equal to d_1_)
$D_{2}$	the maximum diameter of the formed driven head
$D_{3}$	the final maximum diameter of the driven head after elastic recovery
H	the height of installed driven rivet head (or tail head height)
ΔH	the height of driven head convex structure inserted into the hole
$H_{0}$	the height of uninstalled rivet tail out of the sheet-hole
$H_{1}$	the height of rivet tail when the shank touching sheet-hole wall
$H_{2}$	the formed height of the driven head (equal to *H*)
$H_{3}$	the final height of the driven head after spring-back
L	the length of installed rivet shank
$L_{0}$	the length of original rivet shank
$L_{1}$	the length of rivet shank when the shank touching sheet-hole wall
$L_{2}$	the formed length of rivet shank
$L_{3}$	the formed length of rivet shank after elastic recovery
$d_{1}$	the diameter of original sheet-hole (before rivet shank expansion)
$d_{2}$	the diameter of expanded sheet-hole considering the uniform deformation
$d_{2a}$	the diameter of the bottom of expanded sheet-hole considering the non-uniform deformation
$d_{2b}$	the diameter of the bottom of expanded sheet-hole considering convex structure of the driven head
$d_{3}$	the minimum diameter of the driven head
$d_{5}$	the bottom diameter of the driven head
E	material’s Young’s modulus
e	Euler’s constant (natural number)
$F_{\mathrm{sq}}$	riveting squeezing force
K	sheet material strength coefficient
$K_{r}$	rivet material strength coefficient
n	sheet material strain hardening coefficient as per Hollomon, in which *σ* is equal to *K*(ε)^n^
$n_{r}$	rivet material strain hardening coefficient
T	the thickness of whole sheet
$t_{1}$	the thickness of upper sheet
$t_{2}$	the thickness of lower sheet
$\varepsilon_{\mathrm{sq}}$	squeeze true strain at rivet tail head
μ	friction coefficient (Coulomb friction)
$\sigma_{s}$	rivet squeeze stress in driven head
$\sigma_{y}$	sheet or material yield stress
$\sigma_{\mathrm{yr}}$	rivet material yield stress
